# Microwave Sensor for the Determination of DMSO Concentration in Water–DMSO Binary Mixture

**DOI:** 10.3390/mi14071378

**Published:** 2023-07-05

**Authors:** Supakorn Harnsoongnoen, Benjaporn Buranrat

**Affiliations:** 1 The Biomimicry for Sustainable Agriculture, Health, Environment and Energy Research Unit, Department of Physics, Faculty of Science, Mahasarakham University, Kantarawichai District, Maha Sarakham 44150, Thailand; 2 Faculty of Medicine, Mahasarakham University, Muang District, Maha Sarakham 44000, Thailand; benjaporn.b@msu.ac.th

**Keywords:** microwave sensor, hexagonal complementary ring resonator (HCRR), DMSO–water mixtures, interdigital capacitor, microstrip antenna

## Abstract

This research aims to develop a microwave sensor to accurately measure the concentration of dimethyl sulfoxide (DMSO) in water–DMSO binary mixtures. The proposed sensor will utilize microwave frequency measurements to determine the DMSO concentration, providing a non-invasive and efficient method for analyzing DMSO solutions. The research will involve the design, fabrication, and testing of the sensor, as well as the development of an appropriate calibration model. The outcomes of this study will contribute to improved monitoring and quality control in various fields, including pharmaceuticals, chemical synthesis, and biomedical research. The binary mixtures of dimethyl sulfoxide (DMSO) and water with varying concentrations were investigated in the frequency range of 1 GHz to 5 GHz at room temperature using a microwave sensor. The proposed microwave sensor design was based on an interdigital capacitor (IDC) microstrip antenna loaded with a hexagonal complementary ring resonator (HCRR). The performance of the sensor, fabricated using the print circuit board (PCB) technique, was validated through simulations and experiments. The reflection coefficient (S_11_) and resonance frequency (F_r_) of binary mixtures of DMSO and water solutions were recorded and analyzed for DMSO concentrations ranging from 0% *v*/*v* to 75% *v*/*v*. Mathematical models were developed to analyze the data, and laboratory tests showed that the sensor can detect levels of DMSO/water binary mixtures. The sensor is capable of detecting DMSO concentrations ranging from 0% *v*/*v* to 75% *v*/*v*, with a maximum sensitivity of 0.138 dB/% for S_11_ and ΔS_11_ and 0.2 MHz/% for F_r_ and ΔF_r_ at a concentration of 50% *v*/*v*. The developed microwave sensor can serve as an alternative for detecting DMSO concentrations in water using a simple and cost-effective technique. This method can effectively analyze a wide range of concentrations, including highly concentrated solutions, quickly and easily.

## 1. Introduction

Dimethyl sulfoxide (DMSO), also known as (CH_3_)_2_SO, is a colorless, water-soluble, hygroscopic, slightly alkaline organic non-toxic liquid with a slight odor that boils at 189 °C and freezes at 18.5 °C. DMSO is a widely used solvent in the fields of biology, chemistry, pharmacology, and medicine for various applications [1,2]. Its advantageous properties, including low toxicity and environmental compatibility, make it a highly valuable polar aprotic solvent for a variety of applications. Among its many uses, it serves as a cryopreservation agent for cells, a penetration enhancer in topical treatments, and a vital component in the fields of toxicology and pharmacology [3,4,5]. However, it is known that high concentrations of DMSO are cytotoxic. Therefore, it is important to measure and define threshold concentrations of DMSO for cells [6]. DMSO is often a principal additive in assay buffers, with a concentration normally ranging from 0.1% to 5% [7]. It is generally accepted to be non-toxic at concentrations below 10% [3,4,5]. Nevertheless, it should be noted that excessive concentrations of DMSO above 50% introduced into the bloodstream can lead to hemolysis, while direct intravenous injection of DMSO may cause local irritation and necrosis [8]. Therefore, measuring and monitoring the DMSO concentration is very important. Many methods exist for detecting the DMSO concentration, such as gas chromatography (GC) [9], high-performance liquid chromatography (HPLC) [10], mass spectrometry (MS) [11], near-infrared (NIR) spectroscopy [12], and the organic sulfur sequential chemical analysis robot (OSSCAR) [13]. In addition to measuring the DMSO concentration, research has also been conducted on the behavior resulting from the mixing of DMSO with water. The mixing behavior of the DMSO/water system is widely recognized for its strong non-ideal characteristics. Solutions comprising this solvent mixture deviate significantly from the ideal behavior and manifest physical and chemical properties that deviate from the norm of what is generally anticipated for an ideal solution. The non-ideal characteristics of the DMSO/water system are evident in various physical properties, including viscosity, density, calorimetry, refractive index, hygroscopicity, and the translational and rotational motion of DMSO–water mixtures [14,15,16]. Understanding the interaction between DMSO and water is crucial for elucidating the mechanisms underlying ice-blocking and biological toxicity. Furthermore, the distinct characteristics of DMSO–water mixtures have been extensively explored and analyzed through a variety of methods, including molecular dynamics (MD) simulations [17,18,19,20,21], soft X-ray spectra [22], neutron diffraction [23,24], vibrational spectroscopy [25], dielectric spectroscopy [26], Rydberg electron-transfer spectroscopy [27], a high-pressure spectroscopic probe [28], infrared absorption spectroscopy [29,30], Raman spectroscopy [31], and Fourier-transform microwave spectroscopy [32]. Another method that has gained interest today is the measurement of material properties using microwave sensors, which has many advantages, such as being easy to build, cheap, and small in size. Additionally, the results can be checked and analyzed in real time. The use of microwave sensors for measuring solution concentrations has been confirmed in various research applications, including the determination of concentrations of salt [33,34], sugar [33,35,36,37,38,39], nitrate and phosphate [40,41], alcohol [42], ethanol [43,44,45,46], methanol [47], glycerol [48], metal ions [49], and equol [50]. However, various research reviews have revealed that there has not been a study or proof of measuring the concentration of DMSO mixed with water using a microwave sensor. We are pleased to present the results of our study, which investigated the use of a microwave sensor to measure the DMSO concentration in water at room temperature. Our method is simple, low-cost, and capable of rapidly analyzing a wide range of concentrations, including high-concentration solutions. With easy operation, we tested concentrations from 0% *v*/*v* to 75% *v*/*v*.

## 2. Materials and Methods

### 2.1. Designing and Fabricating Sensors

**Figure 1:**
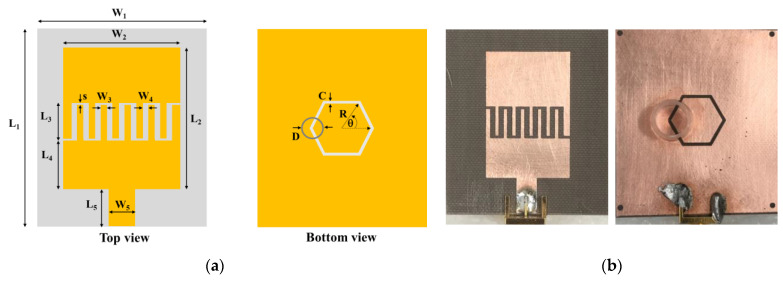
The proposed planar microwave sensor (**a**) layout and (**b**) sensor fabrication.

**Figure 2:**
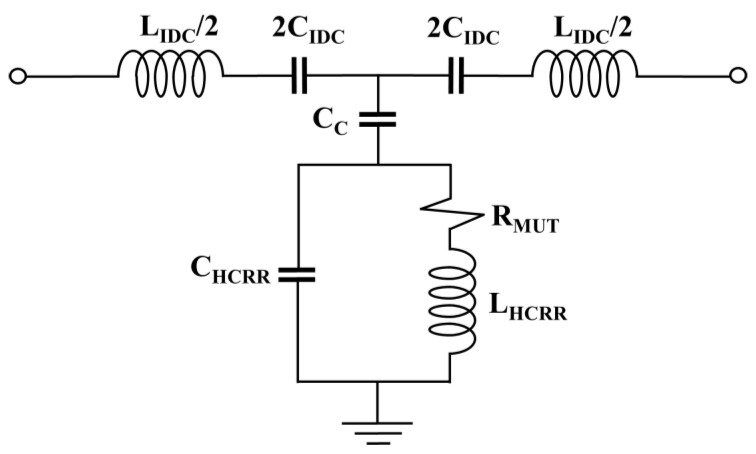
Modeling the proposed sensor using an equivalent circuit.

The proposed microstrip antenna sensor was designed based on an IDC loaded with an HCRR in the ground plane, as shown in Figure 1. The left and right sides of Figure 1a show the IDC and HSRR structures, respectively. A chamber tube is to be equipped in the region of high electric field strength, as shown in Figure 1a on the right side. The fabricated sensor, which is on a DiClad880 substrate with a dielectric constant of ε_r_ = 2.2 and a loss tangent of tan δ = 0.0009 and a thickness of 1.6 mm, is presented in Figure 1b. The layout and dimensions of the sensor structures are shown in Table 1. The equivalent circuit model of the proposed microwave sensor is shown in Figure 2. In our model, the IDC and feed line are represented as a series LC circuit (L_IDC_ and C_IDC_), while the HCRR slot is represented as a parallel C circuit (C_HCRR_) with a series LR circuit (L_HCRR_ and R_MUT_), where R_MUT_ denotes the resistance of the material under test (MUT). The coupling capacitance between the patch with the IDC and the HCRR slot ground plane is represented by C_C_. The equation used to determine the resonance frequency (F_r_) of the proposed device is:

(1) $$F_{r}=\frac{1}{2\pi \sqrt{L_{HCRR}\left(C_{HCRR}+C_{C}\right)}}$$

**Figure 3:**
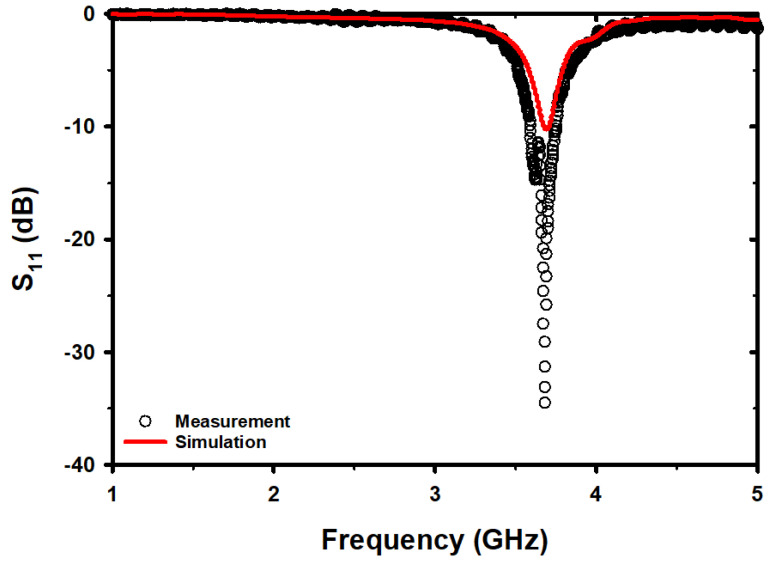
Comparison of simulated and measured S_11_ spectra.

Figure 3 displays a comparison between the S_11_ spectra simulations and measurements for the microwave sensor in free space. The results of the simulations and actual measurements show that the F_r_ is 3.68 GHz and 3.69 GHz, respectively. A frequency difference of 10 MHz represents a 0.27% error. The simulations and actual measurements of S_11_, respectively, have values of −34.5 dB and −10.25 dB. The magnitude of S_11_ in the measurements was found to be lower than that in the simulation, and the reasons behind this discrepancy are not yet fully understood, necessitating further investigation. This discrepancy may be due to parasitic components and differences in size and location during the construction process. This leads to a disparity between the positions of the IDC and HSRR structures in terms of coupling compared to the simulations.

### 2.2. Preparation of Materials and Analyte Solutions

The analytical grade dimethyl sulfoxide (DMSO) that was used in the experiment was purchased from PanReac AppliChem (Cat. No. 67-68-5). Each concentration of DMSO was dissolved in DI water at concentrations of 0, 25, 50, and 75% *v*/*v*. Each concentration of DMSO solution was prepared in triplicate at each concentration.

### 2.3. Experimental Measurement Setup

**Figure 4:**
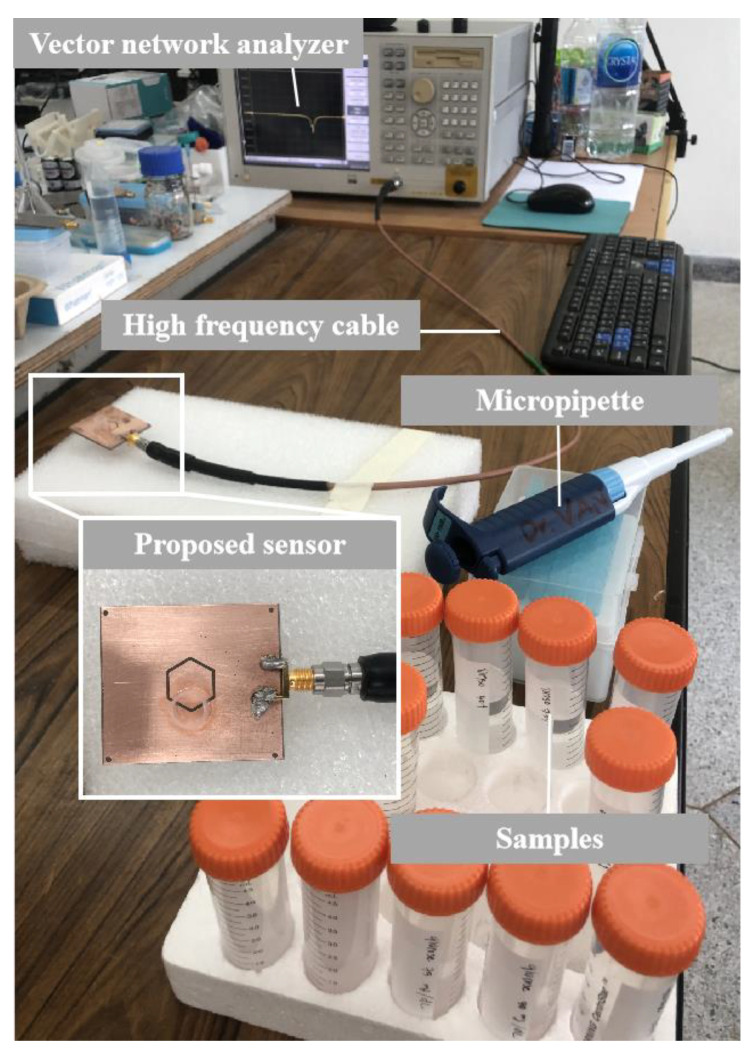
Measurement setup.

Figure 4 illustrates the setup of the sensor and measuring device. The sensor is mounted on a foam base and connected to a Vector Network Analyzer (VNA) via a high-frequency cable. Prior to taking measurements, the short-open-load (SOL) calibration procedure is applied to Port 1 of the VNA. The S_11_ value is subsequently measured and meticulously recorded. The test solution is carefully loaded into the chamber tube using a micropipette. Subsequent to measuring the results, the chamber tube is thoroughly cleansed with DI water after each measurement is completed, ensuring accuracy and reliability. Multiple sample tests, comprising free space, an empty tube, DI water, and varying DMSO concentrations, were conducted. Each measurement was meticulously carried out using 1601 data points within the frequency range of 1–5 GHz to ensure precision and accuracy. The measurement results are plotted to compare the resonance frequency and S_11_ magnitude at different DMSO concentrations and DI water in the frequency range of 3.5–3.75 GHz. The frequency resolution in the measurements is 2.5 MHz, resulting from the 1601 data points used. A total of 0.2 mL of test solution was filled into a chamber tube, the temperature was kept constant at 25 ± 1 °C, and the relative humidity was maintained at 45 ± 1% to ensure a stable and consistent working environment.

## 3. Results

### 3.1. Reflection Coefficient of Sensor

**Figure 5:**
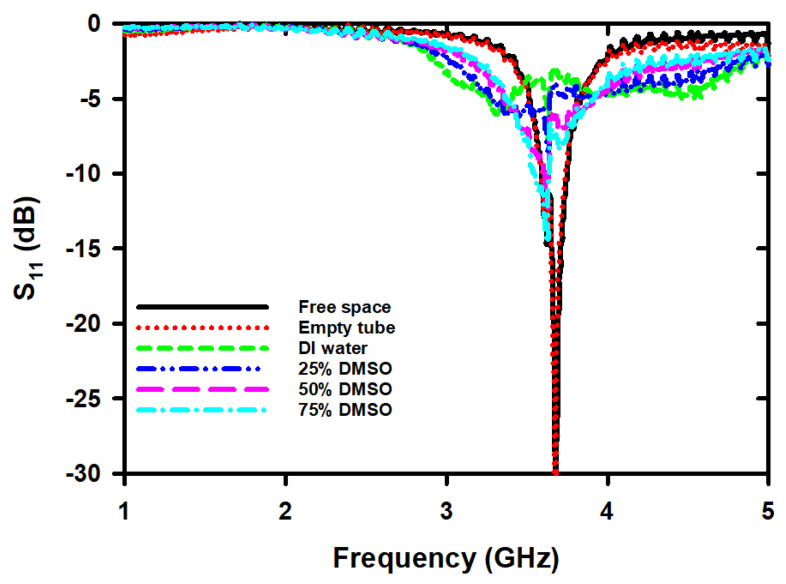
The S_11_ spectra in frequency range of 1 GHz–5 GHz for free space, empty tube, DI water and different concentrations of DMSO/water binary mixture.

**Figure 6:**
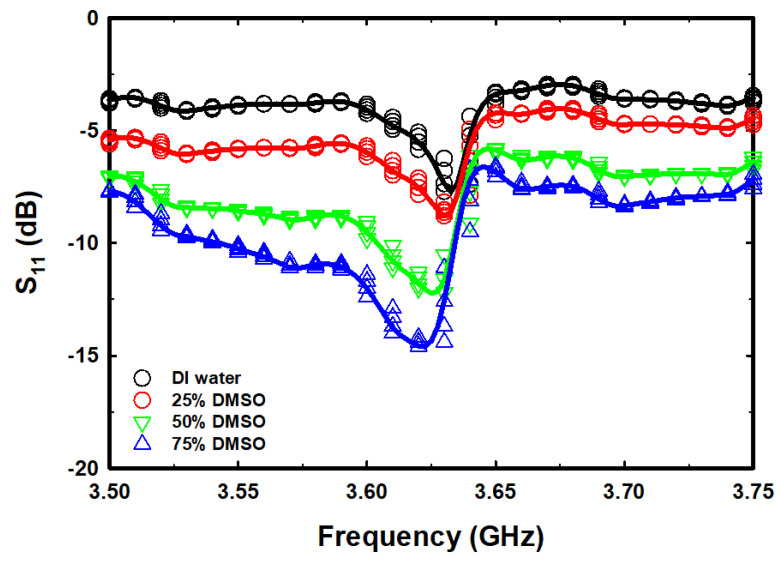
The S_11_ spectra and smoothed data spectra were obtained from measurements of DI water and different concentrations of DMSO/water binary mixture samples versus the 3.5 GHz–3.75 GHz frequency range.

The S_11_ spectra for different sample tests were recorded three times with a time interval of 2 min to ensure stability and reliability. Figure 5 shows the S_11_ spectra for free space, an empty tube, DI water, and different concentrations of DMSO. The magnitude of S_11_ for the sensor with the chamber tube installed has increased by 1.20 dB, and the F_r_ has decreased by 5 MHz compared to the case without the chamber tube installed. When DI water was filled into the chamber tube, the F_r_ dropped from 3.67 GHz to 3.64 GHz, a decrease of approximately 40 MHz, and the magnitude of S_11_ increased from −33.29 dB to −7.70 dB, an increase of 25.59 dB. The decrease in F_r_ is due to the permittivity constant of the DI water being greater than that of air, and the increase in S_11_ is a result of the electrical loss of the DI water being higher than that of air. However, when changing the assay from DI water to various concentrations of DMSO, the F_r_ and S_11_ magnitudes gradually decreased, as shown in Figure 5. We found that the signal of the S_11_ spectra in the frequency range of 3 to 4 GHz was uneven. To clarify the changes, we zoomed in on a narrower frequency range of 3.5 GHz to 3.75 GHz, which covers the notch range of the signal obtained from all the sample tests. Therefore, we applied a smoothing method based on a robust quadratic regression to smooth it out, as shown in Figure 6. However, even though the data was smoothed, the F_r_ and magnitude of S_11_ remained unchanged from the results obtained from the unsmoothed data. 

### 3.2. Linearity of Binary Liquid Mixture Measurement

**Figure 7:**
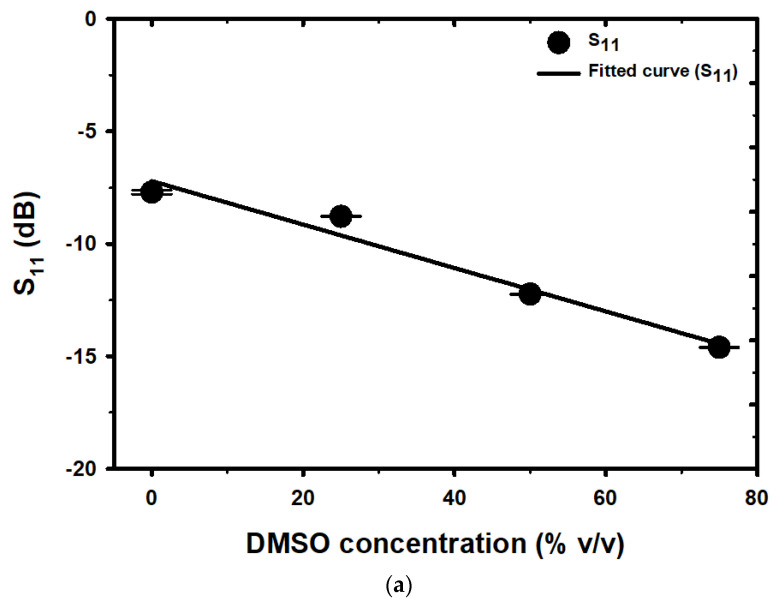

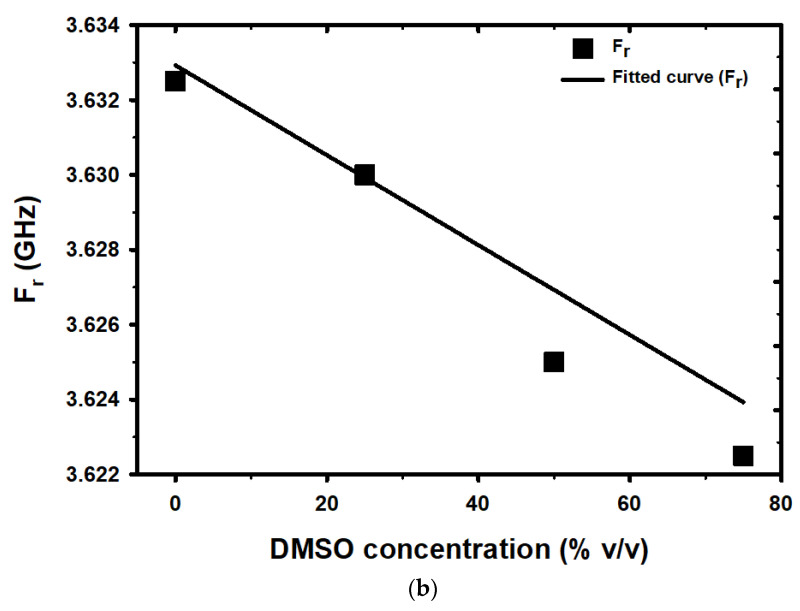
The linearity of (**a**) S_11_ and (**b**) F_r_ with the different concentrations of DMSO.

In this section, we use mixtures of DMSO and water to test the capability of our proposed sensor in characterizing binary liquid mixtures. To accomplish this goal, we carefully prepare DMSO/water mixtures with varying concentrations of 0%, 25%, 50%, and 75% *v*/*v* using a micropipette. This allows us to assess the sensor’s performance in accurately detecting and quantifying the composition of binary liquid mixtures. The magnitude of the S_11_ and F_r_ data at various DMSO concentrations were searched and analyzed to find the relationship between them. The horizontal axis displays the concentration of DMSO, while the left vertical axes show the values of S_11_ and F_r_, as shown in Figure 7a,b, respectively. Scattered data points for both the S_11_ and F_r_ datasets were plotted and fitted to curved trendlines, allowing for the study of the resulting relationship between the variables. The change in S_11_ and F_r_ resulting from the alteration in the concentration of DMSO mixed with water is caused by a variation in the real and imaginary components of the complex permittivity of the DMSO/water binary mixture. The response of the microwave sensor was established by measuring the levels of S_11_ and F_r_ at various concentrations of DMSO. The relationship between the levels of S_11_ and F_r_ at different concentrations of DMSO was then determined using a mathematical model developed through linear regression analysis, which links DMSO concentrations with measurable S_11_ and F_r_ levels. Equation (2) was used to calculate the levels of S_11_ at various DMSO concentrations, which were plotted in Figure 7a. Likewise, the levels of F_r_ at various DMSO concentrations were calculated using Equation (3) and plotted in Figure 7b. The study revealed that the standard deviation (SD) value of F_r_ was lower than that of S_11_, indicating that the F_r_ values were more consistent and less dispersed compared to the S_11_ values.

(2) $$S_{11}\left(dB\right)=-0.0966\rho -7.2069\;\mathrm{with}\;R^{2}\;=\;0.9664$$

(3) $$F_{r}\left(GH_{z}\right)=-14\times 10^{4}\rho +3.63\times 10^{9}\;\mathrm{with}\;R^{2}\;=\;0.9800$$

where ρ represents the concentration of DMSO in units of % *v*/*v*. The relationship between the magnitude of S_11_ and F_r_, obtained from the mathematical model, and the concentration of DMSO in the range of 0–75% *v*/*v* was found to be linear with a negative slope. The results indicated that the magnitude of both S_11_ and F_r_ altered when the concentration of DMSO varied between 0 to 75% *v*/*v*. This step is essential as fluctuations in the complex relative permittivity ($\varepsilon_{r}$) of the sample have the potential to influence the sensor response, which is discernible from the changes in S_11_ and F_r_ measurements. The changes observed in the S_11_ at resonance can be attributed to variations in the sensor impedance, which, in turn, are influenced by the imaginary component ($\varepsilon_{r}^{\prime \prime }$) of the $\varepsilon_{r}$. On the other hand, the change in F_r_ is primarily influenced by variations in C_R_, which is a consequence of changes in the real component ($\varepsilon_{r}^{\prime }$) of the $\varepsilon_{r}$ of the mixed DMSO/water sample. The equation below represents the frequency (f) dependent behavior of the complex relative permittivity for mixed DMSO/water solutions:

(4) $$\varepsilon_{r}\left(f\right)=\varepsilon_{r}^{\prime }\left(f\right)-{j\varepsilon }_{r}^{\prime \prime }\left(f\right)$$

The $\varepsilon_{r}$ can be expressed as a function of the $\varepsilon_{r}^{\prime }$ and the $\varepsilon_{r}^{\prime \prime }$ [21,51,52,53,54,55]. In conducting samples, the $\varepsilon_{r}^{\prime \prime }$ has two contributions, as shown below:

(5) $$\varepsilon_{r}^{\prime \prime }\left(f\right)=\varepsilon_{r_{d}}^{\prime \prime }\left(f\right)-\varepsilon_{r_{\sigma }}^{\prime \prime }\left(f\right)$$

The $\varepsilon_{r}^{\prime \prime }$ is composed of two contributions, namely the dielectric relaxation in dimethyl sulfoxide/loss ($\varepsilon_{r_{d}}^{\prime \prime }$) and the loss due to ion drift ($\varepsilon_{r_{\sigma }}^{\prime \prime }$). At low frequencies, the loss due to ion drift tends to obscure the dielectric contribution of ions.

(6) $$\varepsilon_{r_{\sigma }}^{\prime \prime }=\frac{\sigma }{\varepsilon_{0}2\pi f}$$

Here, σ represents the ionic conductivity, and $\varepsilon_{0}$ denotes the permittivity of free space [55]. The dielectric constant of electrolyte–water solutions can be described by the following equation:

(7) $$\varepsilon_{r}^{\prime }=\varepsilon_{r_{w}}^{\prime }-\alpha c$$

Here, $\varepsilon_{r_{w}}^{\prime }$ denotes the dielectric constant of DI water, c represents the concentration of the electrolyte solution, and α denotes the phenomenological, ion-specific parameter. Figure 7a,b obviously indicate that S_11_ and F_r_ decrease as the concentration of DMSO increases, respectively. It can be inferred that the complex relative permittivity of the mixtures is strongly dependent on the DMSO concentration. This phenomenon is expected to arise from the cooperative motion of DMSO–water molecules through hydrogen bonds [22,55]. As the concentration of DMSO increases within the range of 0–75% *v*/*v*, the $\varepsilon_{r}^{”}$ increases, as reported in [53].

**Figure 8:**
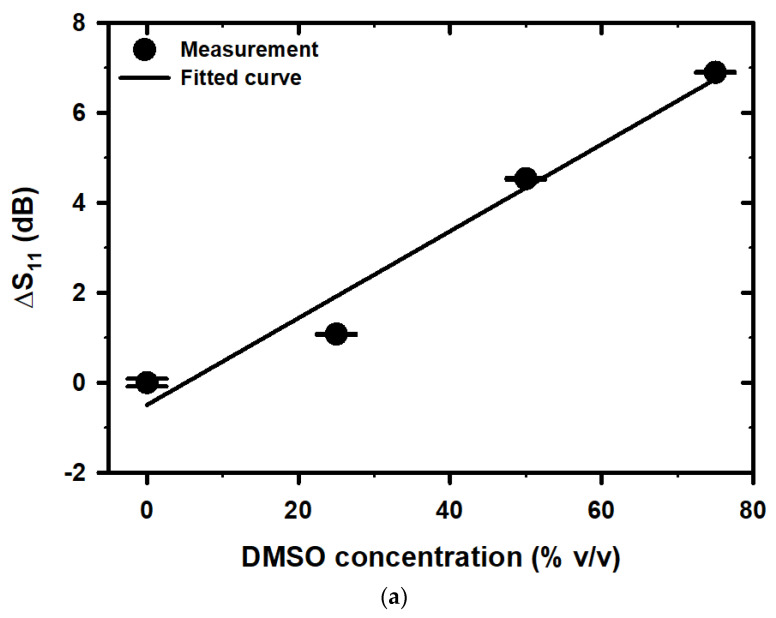

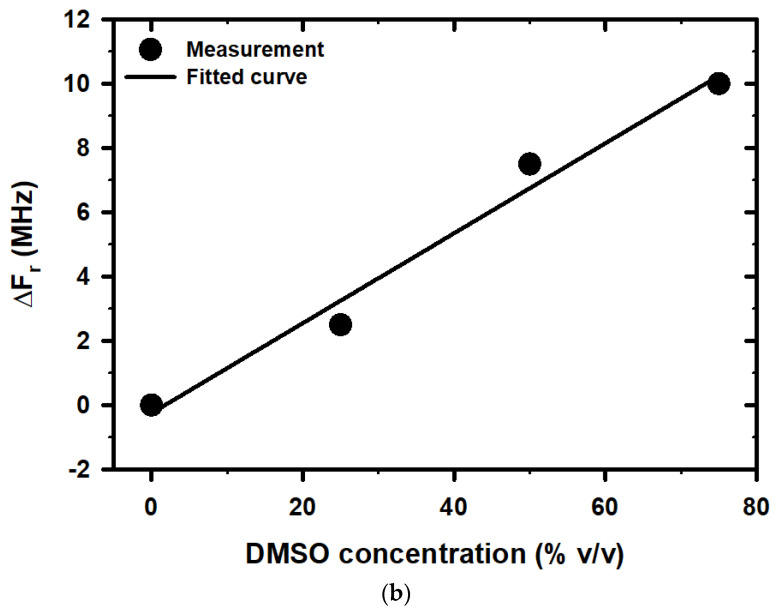
The linearity of (**a**) ΔS_11_ and (**b**) ΔF_r_ with the different concentrations of DMSO.

This study utilized a newly proposed microwave sensor to measure the differences in S_11_ and F_r_ at various concentrations of DMSO and DI water. The resulting data were meticulously analyzed to investigate the relationship between these variables. Figure 8a,b depict the effect of the DMSO concentration on the shift in S_11_ (ΔS_11_) and F_r_ (ΔF_r_) from their reference values, in the range of 0–75% *v*/*v*, using DI water as the reference sample. A linear relationship was found between the $∆S_{11}$ and $∆F_{r}$ and the DMSO concentration, as shown in Equations (8) and (9), respectively.

(8) $$∆S_{11}\left(dB\right)=0.0966\rho -0.4947$$

With R^2^ = 0.9664

(9) $$∆F_{r}\left(MH_{z}\right)=14\times 10^{4}\rho -0.25\times 10^{6}$$

With R^2^ = 0.9800

The S_11_ and F_r_ of the proposed sensor shift upward as the concentration of DMSO in the solution increases. The shift in F_r_ also results in a corresponding change in the S_11_ level at a fixed frequency of 3.64 GHz, which is the F_r_ for DI water. As a result, both S_11_ and F_r_ can be utilized for sensing purposes. The relationship between the S_11_ shift and the DMSO concentration is illustrated in Figure 8a, while the change in F_r_ with respect to the DMSO concentration is depicted in Figure 8b. It was observed that the relationship between the S_11_, F_r_, ΔS_11,_ and ΔF_r_ is linear across the entire range of the 0–75% *v*/*v* change in the DMSO concentration. Furthermore, it was discovered that the R^2^ value for both S_11_ and ΔS_11_ was 0.9664, while the R^2^ value for both F_r_ and ΔF_r_ was even higher at 0.98, indicating a stronger correlation compared to S_11_ and ΔS_11_.

The variations in S_11_ and ΔS_11_ illustrated in Figure 7a and Figure 8a are ascribed to alterations in the losses of binary liquid mixtures at different concentrations. In the context of mixed DMSO/water binary mixtures at microwave frequencies, the losses are from a polar origin, specifically from the reaction of water molecules with the incident field, as calculated using Equations (5) and (6) [56,57]. The variations in F_r_ and ΔF_r_ illustrated in Figure 7b and Figure 8b are ascribed to alterations in the complex permittivity of binary liquid mixtures at different concentrations. To determine the complex permittivity of binary liquid mixtures at varying concentrations, it is possible to employ the dielectric mixture equation, as illustrated in (10) [58,59,60]:

(10) $$\varepsilon_{r}\left(f\right)=\varepsilon_{MUT}\left(f\right)\times \left[\frac{\left({2\varepsilon }_{MUT}\left(f\right)+\varepsilon_{W}\left(f\right)\right)+2V_{VF}\left(\varepsilon_{W}\left(f\right)-\varepsilon_{MUT}\left(f\right)\right)}{\left({2\varepsilon }_{MUT}\left(f\right)+\varepsilon_{W}\left(f\right)\right)-V_{VF}\left(\varepsilon_{W}\left(f\right)-\varepsilon_{MUT}\left(f\right)\right)}\right]$$

where $\varepsilon_{MUT}$ and $\varepsilon_{W}$ are the permittivities of DMSO and DI water, respectively. $V_{VF}$ is the volume fraction of water in the DMSO/water mixtures. This equation can be used to calculate theoretical values of permittivity, providing valuable insights for studying the dielectric behavior of these mixtures. It will enable us to gain a better understanding of the intermolecular interactions and structural properties of liquids, which is significant in various fields of study.

### 3.3. Sensitivity

**Figure 9:**
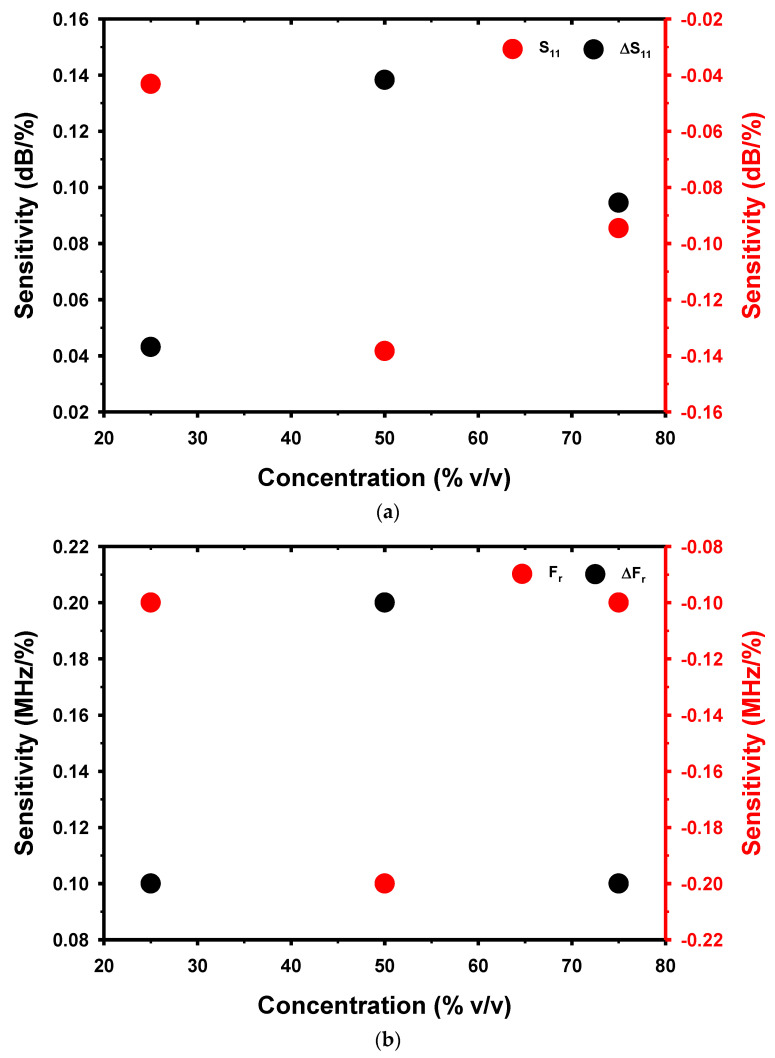
The sensitivity of the sensor and the parameter sensing of (**a**) S_11_ and ΔS_11_ and (**b**) F_r_ and ΔF vary with different concentrations of DMSO.

Figure 9 shows the sensitivity (S) of the proposed microwave sensor obtained from the measurement of the S_11_, F_r_, ΔS_11_, and ΔF_r_ values. The proposed sensor demonstrated an exceptionally high sensitivity when the DMSO concentration was 50% *v*/*v* for the S_11_, F_r_, ΔS_11_, and ΔF_r_ parameters. The reason behind the proposed microwave sensor’s high sensitivity at a 50% *v*/*v* concentration is believed to be that this concentration falls within the range of the DMSO/water mixture, where the imaginary part of the complex permittivity, the Gibbs energy activation, ΔG, and relaxation time are high [53]. The sensitivity calculations, obtained from the S_11_, F_r_, ΔS_11_, and ΔF_r_ values, are presented in Equations (11)–(14), respectively.

(11) $$S_{S_{11}}=\frac{∆S_{11}}{∆\rho }$$

(12) $$S_{F_{r}}=\frac{∆F_{r}}{∆\rho }$$

(13) $$S_{{∆S}_{11}}=\frac{∆(∆S_{11})}{∆\rho }$$

(14) $$S_{∆F_{r}}=\frac{∆(∆F_{r})}{∆\rho }$$

Figure 9a demonstrates that the sensitivity for S_11_ and ΔS_11_ remains consistent across various concentrations of DMSO. However, the sensitivity derived from S_11_ will yield a negative value. Similarly, the sensitivity for F_r_ and ΔF_r_ was found to be consistent across different concentrations of DMSO, with the sensitivity derived from F_r_ being negative, as shown in Figure 9b. When examining the sensitivity derived from all four parameters in Figure 9, it becomes evident that the sensitivity escalates as the DMSO concentration increases until it reaches a maximum at 50% *v*/*v*. Subsequently, the sensitivity gradually diminishes with further increments in the DMSO concentration.

### 3.4. Microwave Sensor Performance for Mixed DMSO/Water Detection

The measurements of the mixed DMSO/water concentration using planar microwave sensors have been presented for the first time. Nevertheless, we conducted a comparative analysis between the concentration range and parameters measured by each technique with the proposed method in order to present a more concise overview of the mixed DMSO/water measurements obtained through different techniques. This analysis is illustrated in Table 2. Unfortunately, due to a lack of available data on the sensitivity of other methods used to measure mixed DMSO/water, we are unable to compare our sensitivity findings. Our study allows for an evaluation of the electrical response of mixed DMSO/water in the microwave frequency range across a wide concentration range, including extremely high concentrations. The analysis of mixed DMSO/water commonly involves a variety of techniques, including GC [9], HPLC [10], MS [11], NIR [12,29,30], OSSCAR [13], soft X-ray [22], dielectric spectroscopy [26], and Raman spectroscopy [31]. GC, MS, and HPLC are renowned for their exceptional performance in measuring mixed DMSO/water, owing to their ability to offer high resolution, specificity, and sensitivity features. These techniques are particularly well suited to analyze low molecular weight compounds and mixtures, making them an excellent choice for such applications. Nevertheless, it is important to note that these methods involve intricate procedures, extended analysis times, frequently require labor-intensive sample pretreatment, and involve costly equipment in addition to necessitating highly specialized technical staff for operation. NIR spectroscopy is a useful and widely used technique for the analysis of mixed solutions, offering several advantages, including speed, efficiency, and non-destructiveness, but also has some limitations, including interferences and the need for specialized instrumentation and expertise. The OSSCAR technique has many advantages for mixed solution measurement, including high selectivity, high sensitivity, and high speed. However, it also has its disadvantages, including complexity, cost, limited scope, and demanding sample preparation. The soft X-ray technique offers several benefits for mixed solution measurement, including high sensitivity, element specificity, and non-destructiveness. Despite these advantages, the technique also has some limitations, including cost, complexity, demanding sample preparation, and limited scope of detection. The equipment required for soft X-ray analysis can be expensive, which may make it difficult for some laboratories to access. Additionally, the techniques involved can be complex and may require specialized technical staff to operate, and the sample preparation process can be demanding and time-consuming, which may impact the accuracy of the results. The advantages of using dielectric spectroscopy for mixed solution measurement include high sensitivity, non-invasiveness, and versatility. The technique can detect small changes in the electrical properties of a sample, making it possible to detect trace amounts of substances, and it does not require any modification of the sample, making it a non-invasive option. Additionally, it can be used for a wide range of applications, including liquids, solids, and suspensions. However, dielectric spectroscopy also has its disadvantages. The analysis of mixed solutions using this technique can be complex and may require specialized technical knowledge. Raman spectroscopy is a highly specific technique for mixed solution measurement, providing molecular-level information without altering the sample. However, its low sensitivity and complexity, as well as the cost and potential requirement for sample preparation, can be disadvantages. Nevertheless, we would like to emphasize that our proposed sensor presents a simple and cost-effective solution that provides rapid analysis, wide concentration measurement capabilities (including high concentrations), and user-friendly operation. In [43,44,45,46,47,48], the sensors exhibit high sensitivity and require a small sample size. However, these sensors necessitate the use of two ports for measurement. Moreover, the integration of microfluidics introduces a complex process of sensor fabrication and assembly. Consequently, the cost of building such sensors is also elevated. In [38], the sensor demonstrates a high level of sensitivity while utilizing only one measurement port. However, such sensors need to be combined with the lump elements. Furthermore, the integration of microfluidics adds complexity to the fabrication and assembly processes of these sensors. As a result, the construction cost of such sensors is also increased. However, the sensitivity of the sensor presented in this study is determined based on measurements of a mixed DMSO/water concentration. Previous studies that utilized microfluidic microwave sensors employed different substances and concentration units, which is frankly inconsistent with the parameters of this study. Consequently, making reasonable comparisons becomes challenging.

## 4. Conclusions

This research presents the design and evaluation of a microwave sensor for measuring DMSO levels in aqueous solutions. The sensor, using an IDC loaded with an HCRR, generates an intense electric field capable of detecting changes in the electrical properties of the specimens. The measured S_11_ spectra and simulation results agreed well within the frequency range of 1 to 5 GHz. The study illustrates a linear correlation between DMSO concentrations and four parameters, namely S_11_, F_r_, ΔS_11_, and ΔF_r_, with maximum sensitivities of 0.138 dB/% (for S_11_ and ΔS_11_) and 0.2 MHz/% (for F_r_ and ΔF_r_), respectively. In conclusion, this research provides a thorough investigation of the microwave sensor’s potential for measuring DMSO levels in aqueous solutions. The proposed method is a simple and cost-effective technique that can effectively analyze a broad range of concentrations, including highly concentrated solutions, in a quick and straightforward manner.

## Figures and Tables

**Table 1:** Layout and geometrical parameters of a microwave sensor.

Parameter	W_1_	W_2_	W_3_	W_4_	W_5_	L_1_	L_2_	L_3_	L_4_	L_5_	S	R	C	D	θ
Value (mm)	40	20	0.8	1.2	5	45	30	7.3	9.6	9.25	0.6	6.4	0.6	8.8	60°

**Table 2:** The comparisons of microwave sensors for DMSO concentration detection.

Ref.	Method	Specimen	Concentration	Data	Sensitivity
[9]	GC	Mixed DMSO/water	0.0001–0.01% *v*/*v*	Voltage	NA
[10]	HPLC	Mixed DMSO/water	0–0.005% *v*/*v*	Absorbance	NA
[11]	MS	Mixed DMSO/ water/NH_3_	0–80 × 10^−12^% *v*/*v*	Phase with solar radiation peaks	NA
[12]	NIR	Mixed DMSO/water	0–20% *v*/*v*	Transmission	NA
[13]	OSSCAR	DMSO/DMSP	0–1.23% *v*/*v*	Voltage	NA
[22]	Soft X-ray	Mixed DMSO/water	0–100% *v*/*v*	Emission intensity	NA
[26]	Dielectric spectroscopy	Mixed DMSO/water	0–47.62% *v*/*v*	S_21_	NA
[29]	Infrared absorption spectroscopy	Mixed DMSO/water	0–0.24% *v*/*v*	Intensity	NA
[30]	Infrared absorption spectroscopy	Mixed DMSO/water	0–47.62% *v*/*v*	Absorbance	NA
[31]	Raman spectroscopy	Mixed DMSO/water	0–90% *v*/*v*	Raman intensity	NA
[38]	Open-ended microstrip transmission line loaded CSRR	Glucose	0−5 mg/mL	$S_{11},F_{r}$	0.5 (dB/(mg/mL)) 0.5 × 10^−3^ (MHz/(mg/mL))
[43]	Microstrip coupled CSRR	Ethanol	0–100% *v*/*v*	$S_{21},F_{r}$	NA
[44]	Microstrip transmission line loaded series LC	Ethanol	0–100% *v*/*v*	$F_{r}$	0.695%
[45]	Microstrip complementary split-ring resonator (MCSRR)	Ethanol	0–100% *v*/*v*	$F_{r}$	0.626%
[46]	Microstrip line loaded CSRR	Ethanol	0–100% *v*/*v*	$F_{r}$	0.98%
[47]	Microstrip transmission line loaded a shunt-connected series LC resonator	Methanol	0–100% *v*/*v*	$S_{21},F_{r}$	0.9%
[48]	Microstrip transmission line terminated with a series RLC resonator	Glycerol	0–90% *v*/*v*	$\left|S_{11}^{DC}\right|$	0.446 (dB/%)
This proposes	IDC loaded HCRR	Mixed DMSO/water	0–75% *v*/*v*	$S_{11},F_{r}$	$0.138\left(dB/\%\right)\;0.2\left(MH_{z}/\%\right)$

NA—data not available.

## Data Availability

Not applicable.
